# Transient Breakage of the Nucleocytoplasmic Barrier Controls Spore Maturation via Mobilizing the Proteasome Subunit Rpn11 in the Fission Yeast *Schizosaccharomyces pombe*

**DOI:** 10.3390/jof6040242

**Published:** 2020-10-23

**Authors:** Hui-Ju Yang, Haruhiko Asakawa, Chizuru Ohtsuki, Tokuko Haraguchi, Yasushi Hiraoka

**Affiliations:** 1 Institute of Molecular and Genomic Medicine, National Health Research Institutes, Zhunan 35053, Taiwan; 2 Graduate School of Frontier Biosciences, Osaka University, Suita 565-0871, Japan; askw@fbs.osaka-u.ac.jp (H.A.); ohtsuki@fbs.osaka-u.ac.jp (C.O.); haraguchi@fbs.osaka-u.ac.jp (T.H.); hiraoka@fbs.osaka-u.ac.jp (Y.H.)

**Keywords:** anaphase-promoting complex (APC/C), leading-edge protein (LEP), meiosis, meu14, forespore membrane, proteasome, rpn11, virtual nuclear envelope breakdown (vNEBD)

## Abstract

Forespore membrane (FSM) closure is a process of specialized cytokinesis in yeast meiosis. FSM closure begins with the contraction of the FSM opening and finishes with the disassembly of the leading-edge proteins (LEPs) from the FSM opening. Here, we show that the FSM opening starts to contract when the event of virtual nuclear envelope breakdown (vNEBD) occurs in anaphase II of the fission yeast *Schizosaccharomyces pombe*. The occurrence of vNEBD controls the redistribution of the proteasomal subunit Rpn11 from the nucleus to the cytosol. To investigate the importance of Rpn11 re-localization during vNEBD, Rpn11 was sequestered at the inner nuclear membrane by fusion with the transmembrane region of Bqt4 (Rpn11-GFP-INM). Remarkably, in the absence of endogenous *rpn11*^+^, the cells carrying Rpn11-GFP-INM had abnormal or no spore formation. Live-cell imaging analysis further reveals that the FSM opening failed to contract when vNEBD occurred, and the LEP Meu14 was persistently present at the FSM in the *rpn11-gfp-*INM cells. The results suggest that the dynamic localization of Rpn11 during vNEBD is essential for spore development.

## 1. Introduction

The fission yeast *Schizosaccharomyces pombe* enters meiosis to form ascospores upon nitrogen starvation [1]. Meiosis involves two sequential nuclear divisions in meiosis I and meiosis II. *S. pombe* undergoes meiotic nuclear divisions without nuclear envelope breakdown. Even though the nuclear envelope remains intact, the nucleocytoplasmic barrier is transiently ineffective at the onset of anaphase II, an event called virtual nuclear envelope breakdown (vNEBD) [2,3]. During vNEBD, the nuclear proteins, except for the integral nuclear membrane proteins, diffuse to the cytosol. The occurrence of vNEBD is actively regulated by the RNA binding protein Spo5, a master regulator of meiosis II progression in *S. pombe*. In the absence of Spo5, the meiotic cells undergo the second nuclear division without vNEBD and fail to form mature spores [2,4,5]. Nonetheless, the causal relationship between vNEBD and spore formation remains undefined. Given that spore development starts in meiosis II, it is tempting to think that the release of the nuclear proteins to the cytosol during vNEBD is involved in the process of spore maturation [6]. 

Spore development starts with de novo biogenesis of the forespore membrane (FSM) within the ascus in meiosis II [7]. The membrane vesicles fuse to form the FSM via the SNARE-mediated membrane fusion events [8]. The FSM grows into a bell-shaped membrane [9]. The lip of a growing FSM is coated by the leading-edge proteins (LEPs), which lead FSM growth along the nuclear envelope [10,11,12,13]. Without the LEPs, the FSM fails to appropriately catch the spore nucleus [12]. After enclosing the spore nucleus, the contraction of the LEP ring initiates FSM closure post-anaphase II [14]. In *S. pombe*, F-actin assembles into the ring structure to consolidate the LEP ring structure and facilitate constriction of the LEP ring. In meiotic cells defective for F-actin assembly, the LEP ring structure is aberrant, and the FSM is overgrown at the site of the FSM leading edge [11]. As FSM closes, the LEP ring dissembles [12]. It is therefore suggested that LEP disassembly is required for the final step of FSM closure. In support of this notion, in the budding yeast *Saccharomyces cerevisiae,* protein degradation of *S. cerevisiae* LEP Ssp1 is required for FSM closure [15]. The Ama1-activated anaphase-promoting complex (APC) is responsible for Ssp1 degradation in anaphase II [16]. The APC is the ubiquitin ligase E3 that tags the substrate proteins with ubiquitin chains. The ubiquitinated proteins are subsequently targeted for 26S proteasome-mediated proteolysis [17]. It remains unclear whether *S. pombe* FSM closure is also controlled by the proteasome-mediated proteolysis of the LEP. 

The 26S proteasome comprises a barrel-shaped core structure and two additional subcomplexes at the lid and the base of the barrel [18]. The core structure harbors the proteolytic activity to degrade the target proteins [18]. Ubiquitination targets the proteins to the proteasome. Before undergoing proteolysis by the core structure, the target proteins are deprived of the ubiquitin chains by the lid subunit Rpn11 for efficient proteolysis [19]. In proliferating and early meiotic cells in *S. pombe,* the proteasome localizes to the nucleus and the inner side of the nuclear membrane. Noticeably, at the end of meiosis, dramatic re-localization of the proteasomal Rpn11 has been reported [20]. In this study, we found that the nuclear Rpn11 diffuses to the cytosol via vNEBD in anaphase II and further addressed the functional significance of the Rpn11 re-localization. Artificial tethering of Rpn11 at the inner nuclear membrane during vNEBD led to abnormal or no spore formation. Moreover, while the FSM grew properly to surround the nucleus, the LEP at the FSM lip was unable to disappear after anaphase II in the mutant, indicating a failure in FSM closure. Our results suggest that the vNEBD-mediated release of the nuclear proteasome facilitates FSM closure in *S. pombe*. 

## 2. Materials and Methods

### 2.1. S. pombe Cell Growth and Sporulation 

*S. pombe* was grown on yeast extract with supplements of adenine, histidine, leucine, lysine, and uracil (YES) plates or Edinburgh minimal medium (EMM) with the appropriate supplements at 30 °C [21]. For sporulation, cells were freshly grown on YES plates overnight, and resuspension of the overnight culture at a density of 10^9^ cells/mL was spotted on a malt extract (ME) plate. The cells on the ME plates were incubated at 26 °C. Between 8 to 15 h after sporulating on the ME plate, the cells undergoing karyogamy were picked for live-cell imaging. For sporulation frequency, the yeast cells were allowed to sporulate on the ME plate for 3–4 days. 

#### 2.1.1. Plasmid and Strain Construction

The *S. pomb*e strains used in the study are listed in Table 1. The *spo5*Δ strain is derived from the strain HA979 [2]. The *rpn11*Δ strain originated from the diploid strain TP42 (*h^-^/h^+^ leu1/leu1 ura4/ura4 his2/+ +/rpn11::ura4^+^ ade6-M210/ade6-M216*, a gift from Takashi Toda, Hiroshima University, Japan [22]). To visualize Rpn11, a 1927-base pair of genomic DNA fragments containing *rpn11*^+^ 5′UTR and open reading frame (ORF) was cloned into an integrating plasmid with the addition of gene encoding GFP or mCherry at the 3′ end of *rpn11*^+^ [23]. To sequester Rpn11 at the inner nuclear envelope, the fluorescence tag was fused with the amino acid 263–432 of Bqt4 (Bqt4dN) [24]. The *aur1*^r^-integrating plasmids carrying the genes of *rpn11^+^*-*gfp-Bqt4dN* or *rpn11^+^*-*gfp* were introduced in the diploid yeast TP42. The resultant transformants were sporulated and dissected to obtain the haploid *rpn11*Δ cells that were resistant to aureobasidin A (Takara Bio Inc., Kusatsu, Japan). On the other hand, Rpn11 endogenously tagged with GFP was derived from the strain 9184 (a gift from Kathleen L. Gould, Vanderbilt University School of Medicine, Nashville, TN, USA; [25]). The FSM marker was encoded by mCherry-tagged *psy1*^+^ integrated at the *lys1*^+^-locus. Chromosomes were visualized using histone H3 Hht1 tagged with mCherry at the native chromosome locus. A two-step PCR method introducing chromosomal mCherry tag was used to fluorescently label Meu14 and Hht1 [26].

#### 2.1.2. Microscopy

Live-cell imaging analysis was performed using an Olympus inverted microscope IX70 equipped with a CoolSNAP HQ^2^ charge-coupled device as previously described (Photometrics, Tucson, AZ, USA; [27]). Briefly, a touch of the sporulating cells on the ME plate was resuspended in the medium of EMM minus nitrogen with the supplements. The solution was added to a lectin-coated (0.2 mg/mL; lectin from Glycine max, Sigma-Aldrich, Tokyo, Japan) glass-bottom dish (MatTek Corp, Ashland, OR, USA). The cells were allowed to adhere to the glass slide via gravity for 5 min. The cells were observed using the Olympus oil-immersion 60× objective lens (PlanApoN60× OSC; NA = 1.4; Olympus, Tokyo, Japan). Optical section images were acquired at 0.5-μm intervals every 6 min using DeltaVision softWoRx 5.5 (Applied Precision, Inc., Seattle, WA, USA). The images were enhanced by three-dimensional constrained iterative deconvolution in softWoRx 5.5.

## 3. Results

### 3.1. Diffusion of Nuclear Rpn11 during Virtual Nuclear Envelope Breakdown (vNEBD)

**Figure 1:**
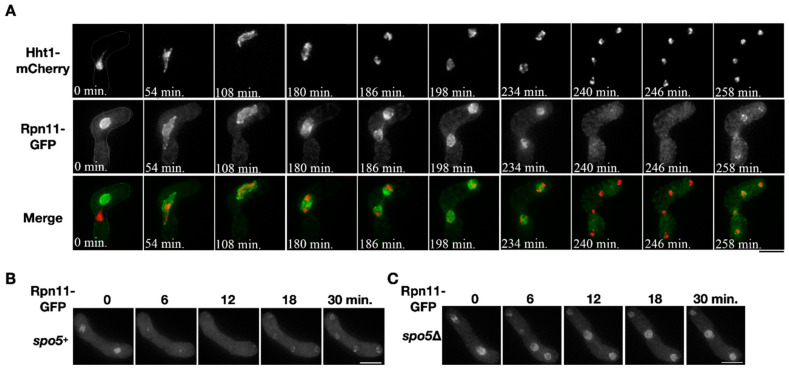
Diffusion of nuclear Rpn11 in anaphase II depends on virtual nuclear envelope breakdown (vNEBD). (**A**) A representative time-course image of the chromosome marker Hht1-mCherry (red) and Rpn11-GFP (green) throughout meiosis in the heterothallic mating cell. (**B**,**C**) Time-course observation of Rpn11-GFP in the wild type or *spo5*Δ mutant during anaphase II. Scale bar: 5 µm.

Localization of the proteasomal subunit Rpn11 was examined during sporulation. Two opposite mating types of the heterothallic haploid cells that respectively expressed Rpn11-GFP and the chromosome marker Hht1-mCherry were mated to enter meiosis and sporulation. Before nuclear fusion, the fluorescence signals of Rpn11-GFP and Hht1-mCherry distinctly labeled each of the two nuclei in the conjugating cell (Figure 1A, time = 0 min). After karyogamy, the two signals merged into a single nucleus where Rpn11-GFP surrounded the chromosome and outlined the nucleus, indicating that Rpn11 localizes at the nuclear periphery and nucleoplasm (Figure 1A, time = 0~108 min). During chromosome segregation in meiosis I, the signals of Rpn11-GFP were enriched around the chromosomes, as previously reported [20] (Figure 1A, time = 180–198 min). On the other hand, the Rpn11-GFP signals shortly disappeared from the nucleus during anaphase II (Figure 1A, time = 240–246 min) but soon re-localized back to the nuclear periphery of the daughter nuclei after completion of the second nuclear division (Figure 1A, time = 258 min).

The transient disappearance of Rpn11-GFP from the nucleus was also observed in anaphase II of the wild-type homothallic sporulating cells (Figure 1B, time = 6–12 min). We investigated whether the disappearance of Rpn11-GFP depends on the occurrence of vNEBD that controls the diffusion of nuclear protein to cytosol at the onset of anaphase II [2]. To do so, we used the *spo5*Δ mutant that undergoes the second nuclear division without vNEBD [2]. In the *spo5*Δ homothallic mutant, the signals of Rpn11-GFP remained in the nucleus at the onset of anaphase II (Figure 1C, time = 6–12 min). The results indicated that nuclear Rpn11 diffuses to the cytosol via vNEBD in sporulating cells.

### 3.2. Retention of Rpn11 at the Inner Nuclear Membrane during vNEBD Causes Abnormal Spore Formation

**Figure 2:**
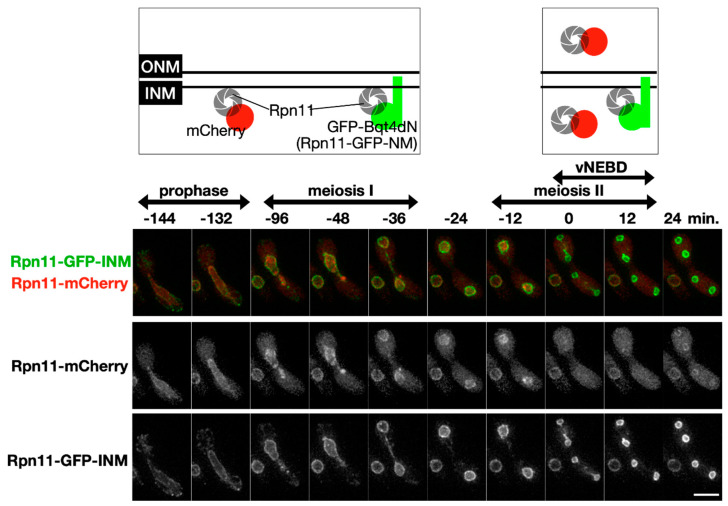
The fusion protein Rpn11-GFP-INM stays at the inner nuclear membrane during vNEBD. Upper panel: illustration of Rpn11-mCherry and Rpn11-GFP-INM localization at different meiotic cell cycle stage. Lower panel: representative time-lapse fluorescence images of Rpn11-mCherry and Rpn11-GFP-INM during meiosis. Scale bar: 5 µm.

It is hypothesized that subcellular re-localization of the nuclear proteins in vNEBD influences spore formation. In an attempt to sequester Rpn11 in the nucleus, we made a fusion construct in which Rpn11-GFP was additionally tagged with the Bqt4 C-terminus (Rpn11-GFP-INM). The C-terminal region of Bqt4 contains the transmembrane domain and inner nuclear membrane targeting sequences [24]. Meiotic localization of Rpn11-GFP-INM was examined in the cells co-expressing mCherry-labeled Rpn11. Unlike the fluorescence signals of Rpn11-mCherry at the nuclear rim and nucleoplasm, the signals of Rpn11-GFP-INM were restricted at the nuclear periphery before meiosis II (Figure 2, time = −144–0 min). Moreover, while Rpn11-mCherry diffused away from the nucleus via vNEBD in meiosis II, the fusion protein Rpn11-GFP-INM remained at the nuclear rim (Figure 2, time = 0–12 min). Thus, Rpn11-GFP-INM successfully sequestered Rpn11 at the inner nuclear membrane during vNEBD.

**Figure 3:**
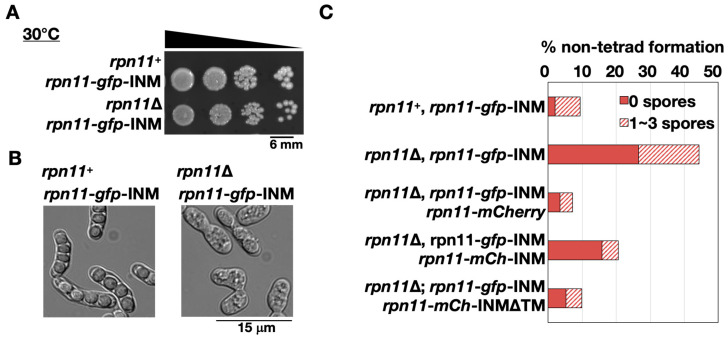
Abnormal ascospore formation in the rpn11-gfp-INM cells. (**A**) Spot assay of the yeast cells’ vegetative growth expressing Rpn11-GFP-INM in the presence or absence of endogenous *rpn11^+^*. The samples (around 10^6^ cells/mL) were 10-fold serial diluted and spotted on the yeast extract with supplement (YES) plate. The plate was incubated at 30 °C for four days. (**B**) Microscopy observation of the yeast sporulation after three days on the malt extract (ME) plate. (**C**) Non-tetrad formation frequency of the cells expressing Rpn11-GFP-INM or co-expressing Rpn11-GFP-INM and the other Rpn11-fusion proteins. Non-tetrad was classified into two groups: the ascus containing 0 spores (solid bar) or 1–3 spores (striped bar). (% of non-tetrad formation = the number of asci containing non-tetrad/the total number of asci).

The fusion construct *rpn11*-*gfp*-INM was introduced to the cells lacking endogenous *rpn11*^+^ to make the homothallic *rpn11*-*gfp*-INM cells (*rpn11*Δ *rpn11*-*gfp*-INM). The *rpn11*-*gfp*-INM cells had no obvious vegetative growth defects at 30 °C (Figure 3A). Because *rpn11*^+^ is an essential gene, the results indicate that Rpn11-GFP-INM is functional for vegetative growth. Next, we examined the ability of cells to undergo sporulation by quantifying the tetrad formation frequency. *S. pombe* normally forms four spores per ascus (tetrad). In the presence of endogenous *rpn11*^+^, around 90% of the zygotic cells expressing Rpn11-GFP-INM formed the tetrads (*rpn11^+^ rpn11*-*gfp*-INM in Figure 3B,C). In contrast, more than 40% of the *rpn11*-*gfp*-INM zygotic cells gave rise to the non-tetrad formation (*rpn11*Δ *rpn11*-*gfp*-INM in Figure 3B,C). In particular, over two-thirds of the non-tetrad in the *rpn11*-*gfp*-INM mutant showed no spore formation (Figure 3B,C). We reasoned that the dynamic localization of Rpn11 is vital for its full function in tetrad formation. To test this idea, we performed a rescue experiment. Adding extra copies of genes expressing Rpn11-mCherry in the *rpn11-gfp*-INM cells fully restored tetrad formation frequency comparable to the wild-type level. By contrast, the mutant cells expressing Rpn11-mCherry fused with the Bqt4 C-terminus (Rpn11-mCh-INM) still had a significant ratio of no spore formation. The Bqt4 transmembrane region of the Rpn11-mCh-INM was then removed to allow Rpn11 mobilization (Rpn11-mCh-INMΔTM). Strikingly, the moveable Rpn11 fusion protein expression in the *rpn11*-*gfp*-INM cells gave rise to the wild-type level of tetrad formation frequency (Figure 3C). The results suggest that *rpn11*-*gfp*-INM represents a recessive allele for non-tetrad formation and emphasize the important role of moveable Rpn11 in sporulation.

### 3.3. FSM Closure is Defective When Rpn11 Is Confined at the Inner Nuclear Membrane during vNEBD

**Figure 4:**
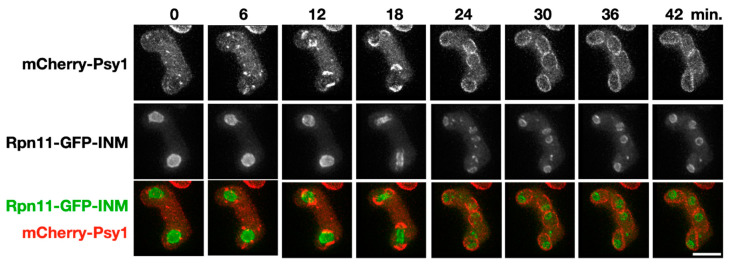
Forespore membrane (FSM) assembly in the *rpn11-gfp-*INM cells. A representative time-lapse image of FSM assembly during meiosis II in the *rpn11-gfp-*INM cells. The FSM is labeled by mCherry-Psy1 (red). The nuclei are outlined by the signal of Rpn11-GFP-INM (green). Scale bar: 5 µm.

Around one-fourth of the *rpn11*-*gfp*-INM zygotic cells failed to form visible spores after three days of sporulation (Figure 3), raising the possibility that the mutant cells are defective in spore development per se. We therefore monitored the initial event of spore development, i.e., FSM assembly, through time-course microscopy of mCherry-tagged Psy1 in the *rpn11*-*gfp*-INM cells. The t-SNARE protein Psy1 that mediates membrane vesicle fusion at the FSM serves as the FSM marker [8]. As previously reported [9], the FSM precursors appeared as cytosolic punctate structures that coalesced into four arcs near the two meiosis II nuclei before anaphase II (Figure 4, 0–12 min), indicating fusion of the membrane vesicles into the FSM. After anaphase II, the FSM extended along the nuclear envelope to capture the divided nuclei (Figure 4, 18–42 min). We concluded that FSM initiation and extension is normal in the *rpn11-gfp*-INM cells.

**Figure 5:**
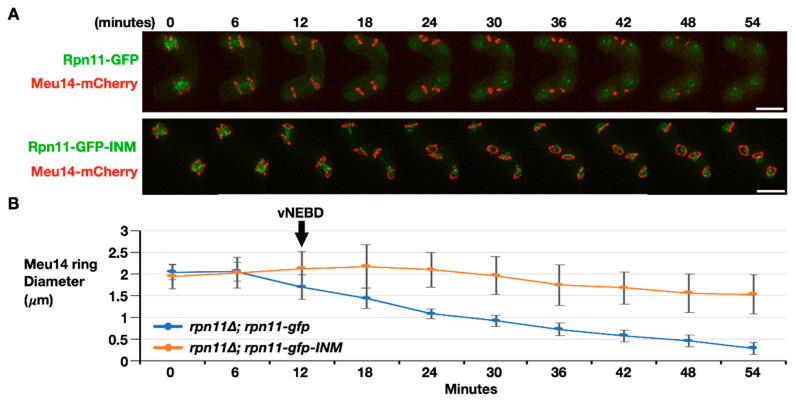
FSM closure is defective in the *rpn11-gfp-*INM cells. (**A**) Representative time-lapse images of FSM closure monitored by dynamics of Meu14-mCherry in the *rpn11*Δ cells expressing Rpn11-GFP (upper panel) or Rpn11-GFP-INM (lower panel). Scale bar: 5 µm. (**B**) Quantification of the Meu14 ring diameter over the time course. At least 30 of the Meu14 rings were tracked and quantified at the indicated time points for each strain. ImageJ was used for quantification. The black arrow indicates the onset of anaphase II when the vNEBD occurs.

The final step of FSM assembly is FSM closure, which separates the spore cytoplasm from the ascus cytoplasm. The fusion protein Meu14-mCherry was used to examine FSM closure dynamics because the LEP protein Meu14 decorates the FSM opening. We first monitored the signals of Meu14-mCherry in the cells expressing Rpn11-GFP as the wild-type control. Before anaphase II, Meu14-mCherry appeared as the ring (or line) structures surrounding the nuclei at the diameter of 2 μm (Figure 5, 0–6 min). Typically, the Meu14 rings’ diameter started to shrink as the nuclear Rpn11-GFP diffused to the cytosol during vNEBD at the onset of anaphase II (Figure 5A upper panel, 12 min). The size of each Meu14 ring continually dropped with an average rate of 0.036 μm per minute until the signals of Meu14-mCherry were undetectable, indicating the closing of the FSM (Figure 5A upper panel, and Figure 5B blue line, 12–54 min). On the contrary, the Meu14 ring size remained constant at the onset of anaphase II, during which Rpn11-GFP-INM stayed at the inner nuclear rim in the *rpn11-gfp*-INM cells (Figure 5A lower panel, 12 min). From anaphase II onward, the diameter of the Meu14 ring slightly decreased but never down to wild-type levels (Figure 5, 12–54 min). Moreover, the signals of Meu14 were persistently present in the ascus cytoplasm of the *rpn11-gfp*-INM cells. Because Meu14 decorates the FSM opening and disappears from the FSM after FSM closure [12], the results suggest that the FSM fails to close when Rpn11 is forced to stay at the nuclear rim during vNEBD.

## 4. Conclusions and Discussion

The role of vNEBD in spore formation has not previously been defined. The current study has identified proteasomal Rpn11 as the vNEBD substrate of which nuclear localization changes to the cytoplasm in anaphase II (Figure 1). Through fusing Rpn11-GFP with the transmembrane domain of the inner nuclear membrane protein Bqt4, we were able to constrain Rpn11 at the nuclear periphery during vNEBD (Figure 2). Many of the cells with sequestered Rpn11 at the nuclear periphery barely had ascospore formation (Figure 3). The FSM closure, but not the FSM growth, was defective in the *rpn11-gfp-*INM mutant (Figure 4 and Figure 5), consistent with the fact that the occurrence of vNEBD is after FSM growth and followed by FSM closure. Therefore, Rpn11 redistribution to the cytosol via vNEBD is important for spore maturation. At this moment, we cannot rule out that a small fraction of Rpn11 goes to the cytoplasm in meiotic prophase and meiosis I. However, we argue that the spo5 mutants whose cytoplasmic proteasome availability is normal before vNEBD still fail to form mature spores [5], suggesting that cytoplasmic proteasome availability in prophase and meiosis I plays little role in spore development.

FSM closure, a unique form of cytokinesis, starts with constriction of the LEP ring after anaphase II. The septation initiation network (SIN) coordinates the anaphase exit and cytokinesis onset [28]. Intriguingly, the *sin* mutant displays belated LEP ring contraction similar to the *rpn11-gfp-*INM mutant [11]. Moreover, several SIN components undergo proteasomal degradation after anaphase II [29]. Proteolysis of the SIN components is inhibited in the *spo6* mutant of which vNEBD fails to occur [29]. Hence, it is possible that the proteasome subunit Rpn11 regulates FSM closure through the SIN signaling pathway.

It is tempting to think that not only Rpn11 but the 26S holoenzyme disperses from the nucleus to the cytosol during vNEBD, given that the proteasome anchor Cut8 [30,31] also diffuses at anaphase II onset (data not shown). Cytoplasmic relocation of the proteasome occurs possibly to obtain access to the cytosolic substrates, such as the LEP at the FSM leading edges. In *S. cerevisiae*, the LEP Ssp1 is targeted towards proteasomal degradation by the Ama1-activated APC/C. The *ama1*Δ mutant whose Ssp1 fails to be degraded has an unsealed FSM and no spore wall [16,32]. FSM closure is a prerequisite for spore wall development [33,34]. Intriguingly, *S. pombe* cells lacking Fzr1 (homolog of *S. cerevisia*e Ama1) barely assemble the spore wall [35]. Moreover, *S. pombe* LEP Meu14 undergoes proteolysis after anaphase II [12]. It remains to be determined whether Meu14 is subjected to proteasomal degradation by Fzr1-activated APC/C. On the other hand, we observed that Meu14 is persistently present when the proteasome subunit Rpn11 is sequestered to the inner nuclear membrane. Thus, proteasomal degradation of the LEP is likely the conserved mechanism underlying FSM closure in both budding and fission yeasts.

## Figures and Tables

**Table 1:** *S. pombe* strain list.

Strain Name	Genotype	Note
HJY175	*h^+^ ade6-M210 leu1 ura4 (*allele unknown*) rpn11::ura4^+^ aur1^r^-rpn11pro-rpn11^+^-GFP*	
RK787	*h^-^ ura4 (*allele unknown*) ade6-216 lys1-131 hht1-mCherry-hph*	
HJY142	*h^90^ ade6-M210 lys1-131 ura4-D18 leu1-32 spo5*Δ*::ura4^+^ rpn11-GFP::kan^r^*	
HJY165	*h^90^ ade6-M216 ura4-D18 leu1-32 rpn11-GFP::kan^r^*	
HJY258	*h^90^ ade6-M216 his2 leu1 ura4 (*allele unknown*) rpn11::ura4^+^ aur1^r^-rpn11pro-rpn11^+^-GFP-Bqt4dN lys1^+^::Prpn11-rpn11-mCherry*	
HJY198	*h^90^ ade6-M210 leu1-32 ura4-D18 aur1^r^-rpn11pro-rpn11^+^-GFP-Bqt4dN*	
HJY197	*h^90^ ade6-M216 his2 lys1-131 leu1 ura4 (*allele unknown*) rpn11::ura4^+^ aur1^r^-rpn11pro-rpn11^+^-GFP-Bqt4dN*	*rpn11*-*gfp*-INM
HJY432	*h^90^ ade6-M216 his2 leu1 ura4 (*allele unknown*) rpn11::ura4^+^ aur1^r^-rpn11pro-rpn11^+^-GFP-Bqt4dN lys1^+^::Prpn11-rpn11^+^-mCherry-Bqt4dNdTM*	*rpn11*-*mCh*-INM TM
HJY479	*h^90^ ade6-M216 his2 leu1 ura4 (*allele unknown*) rpn11::ura4^+^ aur1^r^-rpn11pro-rpn11^+^-GFP-Bqt4dN lys1^+^::Prpn11-rpn11^+^-mCherry-Bqt4dN*	*rpn11*-*mCh*-INM
HJY231	*h^90^ ade6-M216 his2 leu1 ura4 (*allele unknown*) rpn11::ura4^+^ aur1^r^-rpn11pro-rpn11^+^-GFP-Bqt4dN lys1^+^::Pnmt-mCherry-psy1^+^*	
HJY748	*h^90^ ade6-M216 his2 lys1-131 leu1 ura4 (*allele unknown*) rpn11::ura4^+^ aur1^r^-rpn11pro-rpn11^+^-GFP meu14-mCherry-hph*	
HJY749	*h^90^ ade6-M216 his2 lys1-131 leu1 ura4 (*allele unknown*) rpn11::ura4^+^ aur1^r^-rpn11pro-rpn11^+^-GFP-Bqt4dN meu14-mCherry-hph*	
